# Use of the Staple Line Reinforcement Tool to Reduce the Rate and Completely Avoid Anastomotic Leakage in Functional End-to-End Anastomosis

**DOI:** 10.7759/cureus.67450

**Published:** 2024-08-21

**Authors:** Atsushi Hirose, Masahiro Hada, Yoji Nishida, Toru Kurata, Tomoya Tsukada, Koji Amaya, Masahide Kaji

**Affiliations:** 1 Digestive Surgery, Toyama Prefectural Central Hospital, Toyama, JPN

**Keywords:** stapling device, feea, gastroenterological surgery, colorectal surgery, staple line reinforcement, anastomotic leakage, functional end-to-end anastomosis

## Abstract

Background

In our hospital, anastomotic leakage (AL) is observed in approximately 2% of functional end-to-end anastomosis (FEEA) cases annually. It is also usually observed at the staple line of the entry hole closure in several reoperation cases. This study aimed to investigate whether AL would occur in FEEA using a new staple line reinforcement tool, ECHELON ENDOPATH^®^ Staple Line Reinforcement (SLR) (Ethicon, Raritan, NJ, USA).

Methods

A total of 380 patients (400 anastomoses performed from September 2021, when SLR use began, to the end of February 2024) were compared retrospectively, with a total of 459 patients (469 anastomoses performed from April 2019 to August 2021), the same period before SLR was initiated. In the SLR group, ECHELON FLEX^®^ (Ethicon) 60 mm and GST^® ^system (Ethicon) cartridges were used as stapling devices. A p-value of <0.05 was considered statistically significant.

Results

No AL was observed in the SLR group, with a significant difference between the SLR and non-SLR groups (p=0.0021). By anastomotic organ, the AL rate significantly decreased for small intestine-colon anastomosis (p=0.023), but there was no significant difference in small intestine-small intestine anastomosis (p=0.061) or colon-colon anastomosis (p=0.35) between groups.

Conclusion

Reinforcing the staple line using SLR in FEEA may reduce the AL rate. Although AL has not been observed, we will continue to investigate its causes should it occur in the future.

## Introduction

Functional end-to-end anastomosis (FEEA) is a side-to-side anastomosis using a stapling device conceptualized by Steichen et al. in 1968 [1]. It is one of the most common anastomotic methods for small intestine-small intestine anastomosis (SS), small intestine-colon anastomosis (SC), and colon-colon anastomosis (CC). The anastomotic leakage (AL) rate of FEEA is considered low [2,3]. However, at surgical sites where FEEA is used as a reconstruction method, AL often causes serious illness owing to liquid intestinal contents. Accordingly, AL in FEEA should be avoided as much as possible. In our hospital, AL in FEEA is observed in approximately 2% of cases yearly, and AL is observed at the staple line for entry hole closure in several reoperations performed for AL. We felt the need to take some countermeasures for this phenomenon. Meanwhile, Ethicon introduced ECHELON ENDOPATH® Staple Line Reinforcement (SLR) (Ethicon, Raritan, NJ, USA). This sheet-type reinforcement can be used with the cartridges of the Powered ECHELON FLEX® (Ethicon) and GST® system (Ethicon) stapling device. We began using SLR in September 2021 owing to its potential to reduce the AL rate. This study aimed to compare the AL rates between patients who underwent FEEA using SLR and those who did not.

## Materials and methods

We performed a retrospective comparison of anastomosis-related complications, mainly AL, in patients who underwent FEEA for SS, SC, and CC. The results of 400 anastomoses (380 patients who underwent FEEA using SLR from September 2021 to the end of February 2024) were compared with 469 anastomoses (459 patients from April 2019 to August 2021), the same duration before the use of SLR.

All procedures involving human participants in this study followed the ethical standards of institutional or national research committees, the 1964 Helsinki Declaration, and later amendments or comparable ethical standards. All data used in this study manuscript were obtained from an institutional quality database approved by the Ethical Commission of Toyama Prefectural Central Hospital (approval number: 64-37).

Patient condition assessment

The American Society of Anesthesiologists (ASA) physical status (PS) [4] and Charlson Comorbidity Index (CCI) scores [5] were used as simple indices to understand patient status and underlying comorbidities.

Definition of AL

AL was defined as a confirmed diagnosis based on re-operative findings or when gas or stool leakage was observed from an indwelling drain or incisional wound and intestinal contrast was obtained near the anastomotic site on contrast radiography from the drainage tube. The observation period was 30 days postoperatively.

Bowel preparation

For bowel preparation for elective colectomy, the patient was admitted to the hospital two days preoperatively and was given a liquid diet, mechanical bowel preparation with sodium citrate (MAGCOROL Powder) at 15:00, and 5 mL of sodium picosulfate hydrate 0.75% before sleep. The previous day, the patient received enteral nutrition at each meal, 250 mg of kanamycin, and 250 mg of metronidazole QID for chemical bowel preparation. Drinking water was allowed until 24:00. However, in case of colonic stenosis, the patient fasted, and no preoperative bowel preparation was performed. Scheduled small bowel resections were not performed with constant bowel preparation.

Surgical technique

Before discussing reconstructive procedures, preserving blood flow to prevent AL is important. Our basic reconstruction technique with this point is shown in Video 1.

**Video 1:** Our basic technique of FEEA with SLR In order to avoid AL, the technique of mesenteric resection is important to preserve the blood flow to the anastomosed intestine as well as the technique of FEEA itself. In this video, the method of mesenteric resection is also shown. Even in FEEA without SLR, we try to perform mesenteric resection and intestinal anastomosis, as shown in this video. AL, anastomotic leakage; FEEA, functional end-to-end anastomosis; SLR, Staple Line Reinforcement

The Technique of Mesenteric Dissection and Intestinal Resection for Blood Flow Preservation

To ensure blood flow preservation, the intestine was dissected at the site where several vasa recta were dissected toward the reconstructed side of the intestine after the marginal artery was dissected. This ensured that the marginal vessels remained on the side of the reconstructed intestine. This procedure may also allow the preservation of marginal vessels in obese patients with a thickened mesentery, where it is difficult to identify the marginal vessels. Due to the lack of equipment to evaluate intestinal blood flow using ICG fluorescence, we selectively incised the marginal artery to confirm the presence of arterial effusion when we were concerned about blood flow in the marginal artery.

Intestinal resection was performed using a stapling device. When SLR was used, the cartridge was a gold cartridge of the GST® system and SLR was attached to the cartridge. The same cartridges were used for both small intestine and colon. To avoid unnecessary intestinal damage, the exposed intestinal wall was only separated to the extent that it could be dissected. In addition, the angle at which the stapling device was inserted was tilted slightly toward the reconstructed intestine on the contralateral side of the mesentery rather than perpendicular to the intestine during intestinal dissection. This avoids an ischemic zone at the intestinal resection site.

Reconstruction

**Figure 1:**
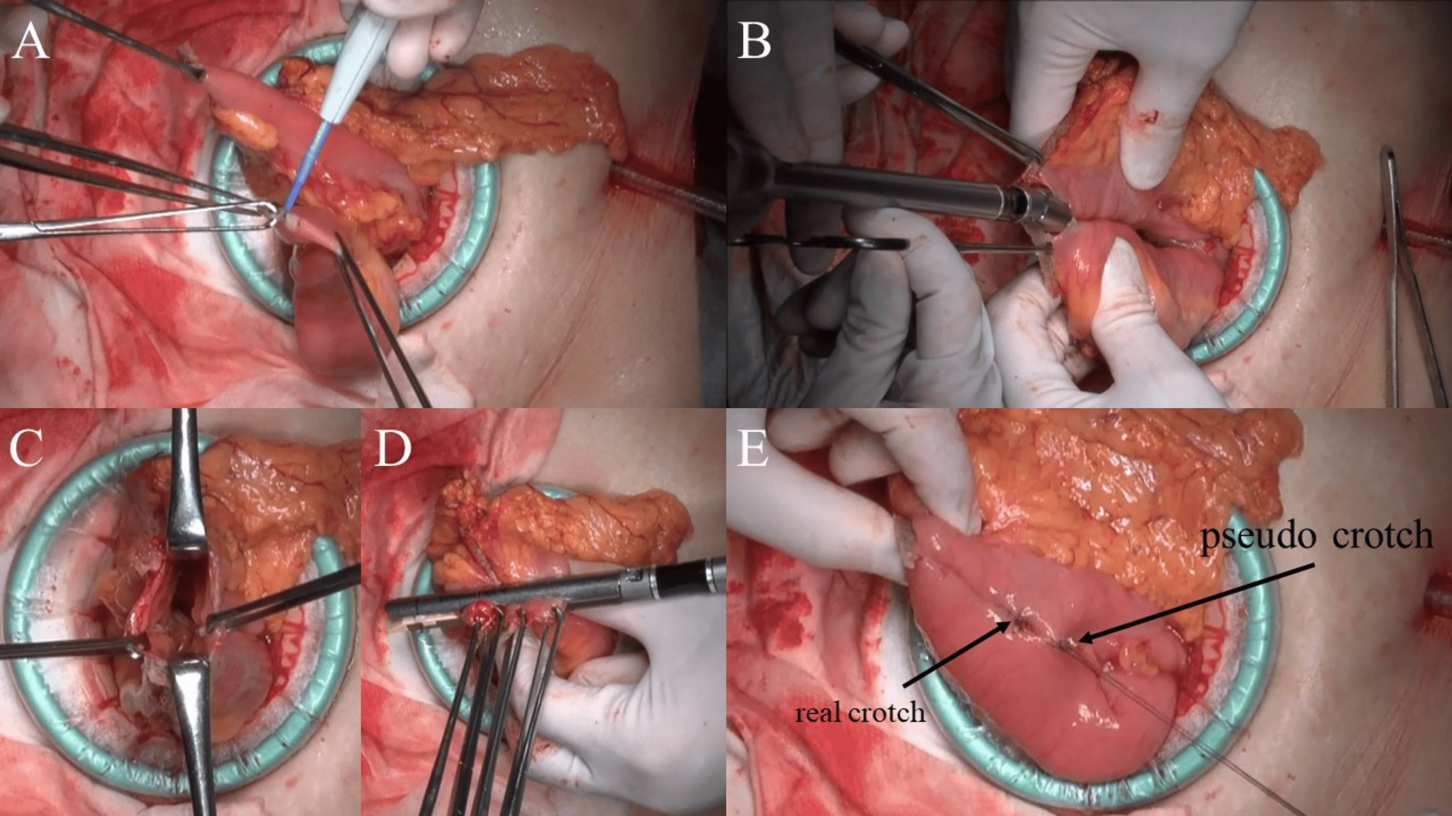
Our technique of functional end-to-end anastomosis with ECHELON ENDOPATH® Staple Line Reinforcement. (A) An incision for inserting stapling device was made along the staple line on the mesenteric contralateral bowel wall. (B) The assistant ensured that the mesenteric contralateral side was the line of suturing the intestinal lumen. SLR cannot be used in the intestinal lumen. (C) Entry hole was grasped so that the anastomosis formed a V-shape. (D) The entry hole was suture closed with Powered ECHELON FLEX® 60mm and gold cartridge of GST® system with SLR attached. (E) Loose serosal muscle layer interrupting suture distal to the real crotch of FEEA as pseudo-crotch to avoid the physical force on the real crotch. FEEA, functional end-to-end anastomosis; SLR, Staple Line Reinforcement

An incision was made along the staple line on the mesenteric contralateral bowel wall to open the bowel lumen (Figure 1A), and a Powered ECHELON FLEX® 60 mm with a gold cartridge of GST® system was inserted. The assistant ensured that the mesenteric contralateral side was the line of dissection (Figure 1B). SLR cannot be used in this firing because it cannot be used in the intestinal lumen. After suturing the intestinal lumen, the entry hole was grasped so that the anastomosis formed a V-shape (Figure 1C). The entire intestinal lumen was securely clamped, and the entry hole was suture closed with a GST® system gold cartridge with SLR attached (Figure 1D). Based on a previous report [6], the crotch of the FEEA was reinforced with a full-layer interrupting suture of 4-0 VICRYL Plus® (Ethicon), and also with a loose serosal muscle layer interrupting suture of 4-0 VICRYL Plus® distal to the crotch. We believe that this area will become a pseudo-crotch and avoid the physical force on the real crotch of the FEEA (Figure 1E). In addition, FEEA was performed using the closed method and extracorporeally in all laparoscopic and robot-assisted laparoscopic surgery cases.

Statistical analysis

Data from the history, physical examinations, laboratory tests, and outcome measures were recorded, entered, and analyzed using Microsoft® Excel® (Microsoft Corporation, Redmond, WA, USA). The data were subsequently imported into IBM® SPSS® (IBM Corp., Armonk, NY, USA) for analysis. The chi-square test and Mann−Whitney’s U test were used to test for statistical significance. A p-value of <0.05 was considered statistically significant.

## Results

Table 1 shows a summary of the results for all organs.

**Table 1:** Summary of anastomosis-related complications in all anastomosis cases *Significant difference between groups (p<0.05)

All anastomosis cases	SLR group (n=400)	No SLR group (n=469)	p-Value
				Anastomotic leakage	0	11 (2.3%)	0.0021*
Anastomotic leakage type of anastomotic organ			
Small intestine-small intestine	0/116	4/135 (3.0%)	0.061
Small intestine-colon	0/196	6/233 (2.6%)	0.023*
Colon-colon	0/88	1/101 (1.0%)	0.35
Bleeding from anastomotic line	1 (0.3%)	0	0.27
Anastomotic stenosis	2 (0.5%)	0	0.13
Intra-abdominal abscess around anastomotic site	1 (0.3%)	2 (0.4%)	0.65
Perianastomotic inflammation	1 (0.3%)	1 (0.2%)	0.91

AL was observed in 11 (2.3%) patients in the non-SLR group but none in the SLR group, indicating that SLR significantly reduced the incidence of AL (p=0.0021). One (0.3%) case in the SLR group and two (0.4%) cases in the non-SLR group had abscesses around the anastomosis. However, these cases were not considered AL because the intestinal lumen was not contrast-enhanced on CT-guided drainage, the drainage was not fecal juice-like, and the patients healed quickly. Since these were all colon resection cases, they were determined to be intracorporeal infections associated with colon resection.

Small intestine-small intestine anastomosis

Table 2 shows the patient background, Table 3 shows the operative factors, and Table 4 shows the results.

**Table 2:** Demographic and preoperative data of the studied groups (small intestine-small intestine anastomosis) ASA PS, American Society of Anesthesiologists Physical Status *Significant difference between groups (p<0.05)

Small intestine-small intestine anastomosis	SLR group (n=116)	no SLR group (n=135)	p-Value
				Sex			
Male	84 (72.4%)	82 (60.7%)	0.051
Female	32 (27.6%)	53 (39.3%)	
Age (years)	69±12	72±13	0.28
Body mass index (kg/m^2^)	22.4±3.8	21.6±3.4	0.15
Primary disease			
Ileus	30 (25.9%)	47 (34.8%)	
Diverting ileostomy closure	33 (28.4%)	39 (28.9%)	
Reconstruction for ileal conduit	18 (15.5%)	13 (9.6%)	
Combined resection for other malignant tumor	6 (5.2%)	12 (8.9%)	
Perforation	9 (7.8%)	5 (3.7%)	
Others	20 (17.2%)	19 (14.1%)	
ASA PSclassification			
I	1 (0.9%)	1 (0.7%)	
II	80 (69.0%)	94 (69.6%)	
III	28 (24.1%)	38 (28.2%)	
IV	7 (6.0%)	2 (1.5%)	
V	0	0	
ASA PS≥3	35 (30.2%)	40 (29.2%)	0.93
Charlson Comorbidity Index scores			
0-1	67 (57.8%)	82 (60.7%)	
2-3	35 (30.2%)	37 (27.4%)	
4-5	2 (1.7%)	7 (5.2%)	
≥6	12 (10.3%)	9 (6.7%)	
Median	1	1	
Previous abdominal surgery			
Yes	53 (45.7%)	83 (61.5%)	0.012*
No	63 (54.3%)	52 (38.5%)	
Chronic steroid treatment	1 (0.9%)	2 (1.5%)	0.65
Serum albumin < 3.5g/dL	23 (19.8%)	36 (26.7%)	0.21
Serum albumin (g/dL)	3.8±0.7	3.7±0.7	0.39
Emergency operation	42 (36.2%)	56 (41.5%)	0.39

**Table 3:** Intraoperative variables in studied groups (small intestine-small intestine anastomosis) *Significant difference between groups (p<0.05)

Small intestine-small intestine anastomosis	SLR group (n=116)	No SLR group (n=135)	p-Value
				Surgical approach			
Open	92 (79.3%)	129(95.6%)	
Laparoscopic	7 (6.0%)	5 (3.7%)	
Robotic assisted	17 (14.7%)	1 (0.7%)	
Operative time (min)	204±203	195±201	0.74
Blood loss (mL, median)	38.5 (0-1990)	50 (0-4304)	
Surgical participants			
Participation of colorectal surgeon	84 (72.4%)	76 (56.2%)	0.008*
Absence of colorectal surgeon	32 (27.6%)	59 (43.8%)	

**Table 4:** Postoperative parameters and anastomosis-related complications (small intestine-small intestine anastomosis)

Small intestine-small intestine anastomosis	SLR group (n=116)	No SLR group (n=135)	p-Value
				Anastomotic leakage	0	4 (3.0%)	0.062
Bleeding from anastomotic line	0	0	
Anastomotic stenosis	0	0	
Intra-abdominal abscess around anastomotic site	0	0	
Perianastomotic inflammation	0	0	
Ileus	9	13 (9.6%)	0.44
Postoperative meal start period (median)	3	4	
Postoperative hospital days (median)	10	11	

In SS, no AL was observed with SLR compared to four (3.0%) patients in the non-SLR group, although the difference was not significant (p=0.062). Two cases of AL (1.5%) were diagnosed with fecal juice in therapeutic intervention, and two cases (1.5%) were diagnosed in reoperation. Nevertheless, significantly fewer patients in the SLR group underwent previous abdominal surgery (p=0.012), and significantly fewer colorectal surgeons participated (p=0.008). All robotic-assisted laparoscopic surgery cases were total cystectomies performed by the urology department.

Small intestine-colon anastomosis

Table 5 shows the patient background, Table 6 shows the surgical factors, and Table 7 shows the results.

**Table 5:** Demographic and preoperative data of the studied groups (small intestine-colon anastomosis) APA PS, American Society of Anesthesiologists Physical Status

Small intestine-colon anastomosis	SLR group (n=196)	No SLR group (n=233)	p-Value
				Sex			
Male	92 (46.9%)	129 (55.3%)	0.081
Female	32 (53.1%)	104 (44.7%)	
Age (years)	73±13	72±13	0.5
Body mass index (kg/m^2^)	22.6±3.4	22.7±3.8	0.98
Primary disease			
Primary colon cancer	159 (81.1%)	189 (81.1%)	
Severe appendicitis	15 (7.7%)	7 (3.0%)	
Combined resection for other malignant tumor	4 (2.0%)	2 (0.9%)	
Others	18 (9.2%)	35 (15.0%)	
ASA PS classification			
I	2 (1.0%)	5 (2.1%)	
II	155 (79.0%)	183 (78.6%)	
III	36 (18.4%)	42 (18.0%)	
IV	3 (1.6%)	3 (1.3%)	
V	0	0	
ASA PS≥3	39 (19.9%)	45 (19.3%)	0.88
Charlson Comorbidity Index scores			
0-1	25 (12.8%)	25 (10.7%)	
2-3	121 (61.7%)	141 (60.5%)	
4-5	23 (11.7%)	31 (13.3%)	
≥6	27 (13.8%)	36 (15.5%)	
Median	3	3	
Previous abdominal surgery			
Yes	72 (36.7%)	75 (32.2%)	0.32
No	124 (63.3%)	158 (77.8%)	
Chronic steroid treatment	2 (1.0%)	7 (3.0%)	0.15
Serum albumin < 3.5 g/dL	60 (30.6%)	70 (30.0%)	0.21
Serum albumin (g/dL)	3.6±0.6	3.7±0.7	0.12
Emergency operation	28 (14.3%)	29 (12.4%)	0.39

**Table 6:** Intraoperative variables in studied groups (small intestine-colon anastomosis)

Small intestine-colon anastomosis	SLR group (n=196)	No SLR group (n=233)	p-Value
				Surgical approach			
Open	47 (24.0%)	70 (30.0%)	
Laparoscopic	146 (74.5%)	163 (70.0%)	
Robotic assisted	3 (1.5%)	0	
Operative time (min)	179±68	180±80	0.39
Blood loss (mL, median)	15 (0–4,614)	23 (0–3,035)	
Surgical participants			
Participation of colorectal surgeon	184 (93.9%)	216 (92.7%)	0.63
Absence of colorectal surgeon	12 (6.1%)	17 (7.3%)	

**Table 7:** Postoperative parameters and anastomosis-related complications (small intestine-colon anastomosis) *Significant difference between groups (p<0.05)

Small intestine-colon anastomosis	SLR group (n=196)	No SLR group (n=233)	p-Value
				Anastomotic leakage	0	6 (2.6%)	0.023*
Bleeding from anastomotic line	0	0	
Anastomotic stenosis	2 (1.0%)	0	0.12
Intra-abdominal abscess around anastomotic site	1 (0.5%)	1 (0.4%)	0.9
Perianastomotic inflammation	1 (0.5%)	0	0.27
Ileus	13 (6.6%)	24 (9.6%)	0.44
Postoperative meal start period (median)	3	3	
Postoperative hospital days (median)	9	11	

In SC, no significant differences were found in patient background or surgical factors. There was no AL in the SLR group, which was significantly lower (p=0.023) than in the non-SLR group (six patients [2.6%]). Three cases of AL (1.3%) were diagnosed with fecal juice from drainage tube that was retained in surgery, but manageable without reoperation. Three cases (1.3%) were required reoperation. Two cases (1.0%) of stenosis in the ileum near the anastomosis were observed in the SLR group. In both cases, conservative treatment did not improve the symptoms, and surgical resection and re-anastomosis were performed. However, pathological examination revealed fibrous stenosis with no characteristic findings.

Colon-colon anastomosis

Table 8 shows the patient background, Table 9 shows the surgical factors, and Table 10 shows the results.

**Table 8:** Demographic and preoperative data of the studied groups (colon-colon anastomosis) APA PS, American Society of Anesthesiologists Physical Status *Significant difference between groups (p<0.05)

Colon-colon anastomosis	SLR group (n=88)	No SLR group (n=101)	p-Value
				Sex			
Male	55 (62.5%)	63 (62.4%)	0.98
Female	33 (37.5%)	38 (37.6%)	
Age (years)	72±12	68±13	0.074
Body mass index (kg/m^2^)	22.4±3.3	21.8±3.3	0.31
Primary disease			
Primary colon cancer	66 (75.0%)	77 (76.2%)	
Combined resection for other malignant tumor	8 (9.1%)	6 (5.9%)	
Diverticula	4 (4.5%)	3 (3.0%)	
Others	10 (11.4%)	15 (14.9%)	
ASA PS classification			
I	3 (3.4%)	8 (7.9%)	
II	66 (75.0%)	73 (72.3%)	
III	17 (19.4%)	19 (18.8%)	
IV	1 (1.1%)	1 (1.0%)	
V	1 (1.1%)	0	
ASA PS≥3	19 (21.6%)	20 (19.8%)	0.76
Charlson Comorbidity Index scores			
0-1	11 (12.5%)	15 (14.9%)	
2-3	45 (51.1%)	62 (61.4%)	
4-5	15 (17.0%)	10 (9.9%)	
≥6	17 (19.4%)	14 (13.8%)	
Median	3	2	
Previous abdominal surgery			
Yes	34 (38.6%)	38 (37.6%)	0.89
No	54 (61.6%)	63 (62.4%)	
Chronic steroid treatment	3 (3.4%)	2 (2.0%)	0.54
Serum albumin < 3.5 g/dL	18 (20.5%)	19 (18.1%)	0.77
Serum albumin (g/dL)	3.7±0.6	3.9±0.5	0.059
Ileus	5 (5.7%)	0	0.015*
Emergency operation	28 (14.3%)	29 (12.4%)	0.39

**Table 9:** Intraoperative variables in studied groups (colon-colon anastomosis)

Colon-colon anastomosis	SLR group (n=88)	No SLR group (n=101)	p-Value
				Surgical approach			
Open	28 (31.8%)	39 (38.6%)	
Laparoscopic	59 (67.0%)	62 (61.4%)	
Robotic assisted	1 (1.2%)	0	
Operative time (min)	205±98	189±64	0.62
Blood loss (mL, median)	21 (0–1,947)	31 (0–1,295)	
Surgical participants			0.3
Participation of colorectal surgeon	77 (87.5%)	93 (92.1%)	
Absence of colorectal surgeon	11 (12.5%)	8 (7.9%)	

**Table 10:** Postoperative parameters and anastomosis-related complications (colon-colon anastomosis) *Significant difference between groups (p<0.05)

Colon-colon anastomosis	SLR group (n=88)	No SLR group (n=101)	p-Value
				Anastomotic leakage	0	1 (1.0%)	0.35
Bleeding from anastomotic line	1 (1.1%)	0	0.28
Anastomotic stenosis	0	0	
Intra-abdominal abscess around anastomotic site	0	0	
Perianastomotic inflammation	0	1 (1.0%)	0.27
Ileus	9 (10.2%)	3 (3.0%)	0.041*
Postoperative meal start period (median)	3	3	
Postoperative hospital days (median)	9	11	

In CC, no AL was observed with SLR, but only one (1.0%) case of AL was observed in the non-SLR group, and the difference was not significant (p=0.35). This case required reoperation. However, there were significantly more ileus cases in the SLR group, and although there were no significant differences, ASA PS ≥3, emergency surgery, and chronic steroid treatment were more common, and serum albumin and participation of lower colorectal surgeons were less common. Anastomotic bleeding on day 1 postoperatively (1.1%) was observed in the SLR group.

## Discussion

Owing to its simplicity, FEEA is one of the most commonly used anastomotic methods in SS, SC, and CC. FEEA has become common because it is not affected by differences in the diameter of the anastomosed intestine, has a large anastomotic diameter, and is relatively easy to perform quickly, regardless of the surgeon’s skill. With the advent of FEEA, there have been very few instances of hand-sewn anastomosis.

The AL rate of FEEA is reportedly low, ranging from 0.19% to 2.49% [2,3]. While there are reports of lower AL rates with FEEA than with hand-sewn anastomosis [4], there are also recent reports of lower AL rates with hand-sewn anastomosis [7,8]. This may be partly due to the surgeon’s skill, and it has been reported that colorectal surgeons have a lower AL rate for FEEA than for general surgeons [9]. Patient-related factors that may cause AL include preoperative malnutrition [10], preoperative albumin level of ≤3.5 g/dL [11-13], ASA score of ≥3 [11,14,15], prolonged surgery [16], emergency surgery [14,17], chronic steroid use [16,18], higher CCI score [19-21], and so on. In contrast, Caziuc et al. reported that local factors such as the surgeon’s technique and blood flow are important surgery-related factors [22]. On the other hand, AL is reported to have a negative impact on overall survival, disease-free survival and local recurrence in cancer surgery [23,24]. In addition, avoidance of AL may lead to shorter hospital stays and lower medical costs. For these reasons, AL should be avoided as much as possible.

**Figure 2:**
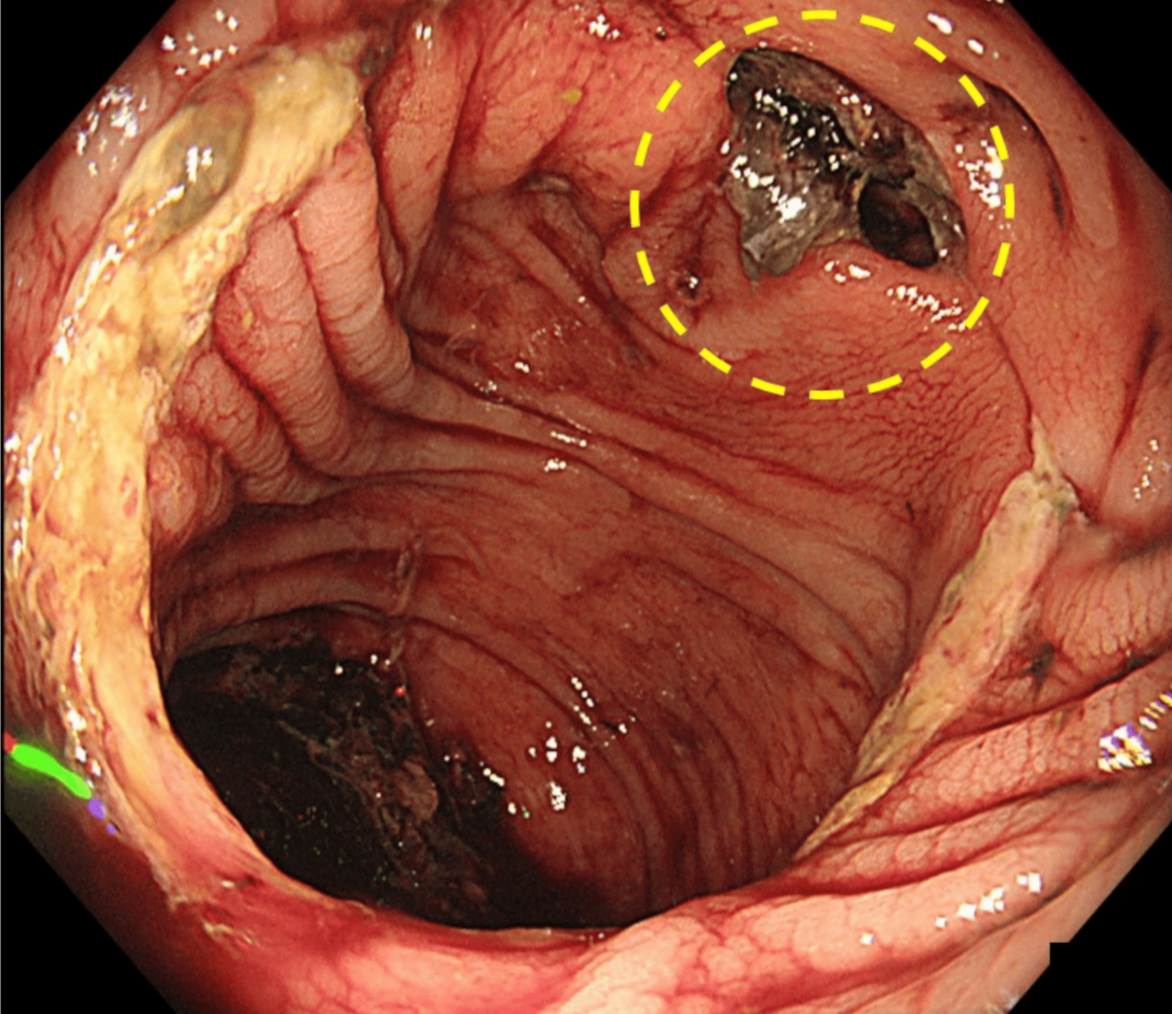
The reoperation case for anastomotic leakage of FEEA without SLR The figure shows the anastomosis at colonoscopy. AL is observed on the contralateral side of V-shaped suture line (circle). FEEA, end-to-end anastomosis; SLR, Staple Line Reinforcement

We encountered several reoperation cases of AL in FEEA that occurred at the site of the staple line used for entry hole closure (Figure 2).

**Figure 3:**
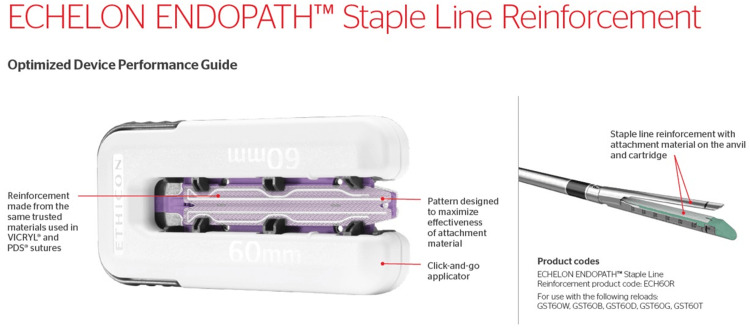
Outline of ECHELON ENDOPATH® Staple Line Reinforcement ECHELON ENDOPATH® Staple Line Reinforcement is a three-layered sheet reinforcement material consisting of a mesh of the same material as VICRYL® (material: polyglactin 910) mesh, laminated on both sides by a PDS® (material: polydioxanone) film used to reduce friction with the tissue to achieve both approachability and reinforcement effect. This device is not recommended for use in the intestinal lumen because of VICRYL® mesh. Easy to use, just clamp this device with the Powered ECHELON FLEX® 60mm GST® system.

Based on this experience, we thought that physical reinforcement of the staple line was important and decided to use ECHELON ENDOPATH® Staple Line Reinforcement (Figure 3) that can be used with GST® system cartridge for Powered ECHELON FLEX®, a stapling device from Echelon Corporation.

SLR is a three-layered sheet reinforcement material consisting of a mesh of the same material as VICRYL® (Ethicon) mesh, laminated on both sides by a PDS® (Ethicon) film used to reduce friction with the tissue to achieve both approachability and reinforcement effect. It should be noted that this sheet is not recommended for use in the intestinal lumen because it is made of VICRYL® mesh and cannot be used for anastomosis between the intestinal tubes in FEEA. We used SLR with the 60 mm GST® gold cartridge for intestinal resection and entry hole closure in FEEA. Not only does this sheet reinforce the staple line, but we also think it is advantageous because there is no tissue contusion at the staple line, which may be caused by the stapler blade spilling, compared to cases without the sheet.

In this study, AL was not observed with the use of SLR, and this difference was significant compared to the non-use group. By anastomotic organ, AL was significantly reduced in SC. Regarding SC, there were no significant differences in patient background or surgical factors, suggesting that SLR is likely to contribute to the reduction of AL. In SS, the use of SLR tended to decrease AL, but there was significantly less previous abdominal surgery in the SLR group. Three of the four AL cases of SS in the non-SLR group included cases of firm adhesions, suggesting that a history of abdominal surgery may have influenced the development of AL. In CC anastomosis, there was no significant difference in AL reduction with the use of SLR. However, the SLR group had worse patient conditions and surgical factors, and SLR may have been responsible for preventing AL.

This study is the first to examine the presence of AL in FEEA using SLR. Although non-colorectal surgeons and residents performed the cases, and not all patients were in good condition, as some had multiple factors impairing tissue healing, AL has not been observed with the use of SLR, and its occurrence was significantly reduced in some anastomotic organs. Recently, the importance of evaluating blood flow to cause AL using indocyanine green (ICG) fluorescence imaging has been reported [25-27], and the evaluation of blood flow using ICG fluorescence is simple and should be performed if possible. However, the present study did not include any cases in which blood flow was evaluated by ICG due to lack of equipment. In addition to basic blood flow preservation techniques, bowel resection, and reconstructive procedures, using SLR with the powered ECHELON FLEX® and GST® system, which allows for easy physical reinforcement, may reduce AL. Furthermore, with the spread of robot-assisted surgery, intracorporeal anastomosis has been reported especially in colon cancer surgery [28-30]. However, there are reports that intracorporeal anastomosis carries a risk of infectious complications [30], and the long-term results including peritoneal dissemination recurrence are not certain in the case of colon cancer. If AL can be avoided as in the present study, we believe that extracorporeal FEEA is an anastomosis method that should be performed aggressively.

This study had some limitations, including the retrospective and historical bias, single-center design, failure to evaluate confounding factors, and a relatively small sample size. The extent of peritonitis, which may also affect anastomotic healing, was not evaluated in emergency operation. Additionally, the number of surgeons is not constant, and many surgeons are involved in the study. However, no AL occurred in the SLR group, and this result may have indicated the usefulness of SLR. We would like to consider a prospective multicenter study.

## Conclusions

Our findings suggest that using SLR, a reinforcement of the staple line of the stapling device, could reduce AL occurrence compared to cases without SLR. We hope to include more cases in the future when AL occurs so that we can investigate the cause in greater detail.
